# Impact of meditation‐only interventions on university students' resilience: A pairwise and network meta‐analysis of randomised actively and passively controlled trials

**DOI:** 10.1111/aphw.70152

**Published:** 2026-06-07

**Authors:** Robin Jacob, Lorena Cascant Ortolano, Theresa Dicks, Pascal Kemmerer, Jennifer L. Reichel, Viktoria Eggert, Thomas Rigotti, Pavel Dietz

**Affiliations:** ^1^ Institute of Occupational, Social and Environmental Medicine University Medical Center of the Johannes Gutenberg University of Mainz Mainz Germany; ^2^ Departmental library University Medical Center of the Johannes Gutenberg University of Mainz Mainz Germany; ^3^ Institute of Medical Biostatistics, Epidemiology and Informatics (IMBEI) University Medical Center of the Johannes Gutenberg University of Mainz Mainz Germany; ^4^ Corporate Health Management, Human Resources and Legal Affairs Technical University of Darmstadt Darmstadt Germany; ^5^ University Cancer Center Mainz (UCT Mainz) University Medical Center of the Johannes Gutenberg University of Mainz Mainz Germany; ^6^ Leibniz Institute for Resilience Research Mainz Germany; ^7^ Department of Work, Organizational, and Business Psychology, Institute for Psychology Johannes Gutenberg University of Mainz Mainz Germany

**Keywords:** mindfulness, network meta‐analysis, psychological resilience, RCT, university students

## Abstract

University students are at elevated risk of psychological distress, underscoring the need for interventions that promote resilience. Psychological resilience refers to the capacity to withstand or recover from stress. This systematic review and meta‐analysis evaluated the effects of meditation‐only interventions on resilience in university students compared with active and passive controls and examined whether effects varied by meditation style, intervention intensity, delivery mode or follow‐up length. A literature search of MEDLINE, PsycINFO and Web of Science identified randomised controlled trials involving university students who participated in meditation‐only interventions and reported resilience‐related outcomes. Pairwise random‐effects models, mixed‐effects meta‐regression and network meta‐analysis were used to estimate standardised mean differences (SMDs) at postintervention. Eighty‐eight studies with 8728 participants were included. The overall effect was −0.11 (95% CI = −0.63 to 0.41, *I* ^2^ = 59%) for resilience scales, 0.27 (95% CI = 0.20–0.35, *I* ^2^ = 40%) for resilience‐related mental health outcomes and 0.54 (95% CI = 0.34–0.73, *I* ^2^ = 76%) for resilience factors. These findings suggest that meditation‐only interventions may improve resilience‐related mental health outcomes and resilience factors, but evidence for direct effects on resilience scales remains limited. Interpretation is constrained by heterogeneity and limited power in moderation analyses.

## INTRODUCTION

There is evidence that the number of students in higher education is increasing (Satilmis, 2019). As university students represent the future workforce of leaders and innovators, they constitute an important segment of society. At the same time, they are exposed to a range of stressors. In particular, students may face academic, social and financial pressures, especially at the beginning of their studies (DeRosier et al., 2013). These factors may increase the risk of mental health problems such as psychological distress, depression and anxiety among university students (Bayram & Bilgel, 2008). The COVID‐19 pandemic has imposed additional burdens for university students, including social distancing, prolonged time at home and e‐learning (Gewalt et al., 2022). These challenges may have further worsened students' mental health (e.g. hopelessness, loneliness, sadness, depression, anxiety and anger; Wood et al., 2024). Furthermore, high stress levels related to physical factors, sleep‐related, emotional strain and personal habits have also been found to contribute to the deterioration of students' mental health (Ashok Kumar et al., 2022). Given the important role of university students in society, the high prevalence of stress in this population and the substantial impact of mental health problems, there is a clear need to protect this vulnerable group from adversity. One important avenue for protection may be the strengthening of psychological resilience.

A systematic review by Chmitorz et al. (2018) highlights that, over the past two decades, the conceptualisation of resilience has shifted from a trait‐oriented perspective to outcome‐ and process‐oriented approaches. Whereas the trait‐oriented perspective conceptualises resilience as a relatively stable personality characteristic, the outcome‐ and process‐oriented perspectives define it in relation to the presence of stressors. In this view, resilience is understood either as a positive outcome following adversity or as the process of adaptation and recovery over time. In particular, the process‐oriented approach emphasises the trajectory of recovery in response to stressors. Chmitorz et al. (2018), and more recently Arnold et al. (2023), also argue that there is currently no gold standard for the assessment of psychological resilience. As a result, resilience is often operationalised using self‐report resilience scales or surrogate outcomes, such as resilience factors and mental health‐related constructs including well‐being, quality of life and subjective stress (Chmitorz et al., 2018).

Most resilience scales appear to assess relatively stable personality traits (e.g. Resilience Scale: RS; Wagnild & Young, 1993) or specific resilience‐related factors, such as social support or self‐efficacy, that may help individuals maintain or regain mental health in the face of adversity (e.g. Connor‐Davidson Resilience Scale: CD‐RISC; Connor & Davidson, 2003). As such, these measures do not assess resilience as an outcome but rather capture presumed protective factors that may facilitate positive adaptation to stress and adversity. By contrast, the Brief Resilience Scale (BRS; B. W. Smith et al., 2008) appears to be one of the few instruments designed to assess resilience more directly as the ability to bounce back or recover from stress. Consistent with this view, Chmitorz et al. (2018) advocate an outcome‐oriented definition of resilience, particularly in the context of intervention research. Several interventions aimed at strengthening students' resilience are discussed in the following section.

Drawing on four systematic reviews and meta‐analyses, Linz et al. (2020) concluded that resilience interventions can improve resilience in adults, with effect sizes ranging from small to moderate. In the systematic review by Joyce et al. (2018), existing resilience interventions were grouped into three categories: (1) mindfulness‐based interventions, (2) cognitive behavioural therapy‐based interventions and (3) mixed interventions. Across these categories, most interventions included combinations of psychoeducation, mindfulness, cognitive skills training, self‐compassion, gratitude practice, emotion regulation training, relaxation and goal setting. Notably, eight out of the 11 studies included by Joyce et al. (2018) evaluated either mindfulness‐based interventions or mixed interventions incorporating mindfulness components. A similar strong representation of mindfulness‐based approaches has been reported in other systematic reviews of resilience interventions (Ang et al., 2022; Fox et al., 2018). Comparable findings have also been observed in university student samples, where resilience interventions involving positive psychology, cognitive behavioural therapy and mindfulness have generally produced small to moderate effects (Enrique Roig et al., 2020; Hechanova et al., 2023; Houston et al., 2017). Taken together, these findings suggest that mindfulness may play a central role in resilience interventions. Supporting this interpretation, Liu et al. (2022) reported a statistically significant meta‐analytic correlation between mindfulness and resilience in university students (*k* = 20, *r* = 0.465). Considering practices grounded in mindfulness principles that may also enhance resilience as an outcome points to meditation as a particularly relevant technique (Montero‐Marin et al., 2016; Tanner et al., 2009).

According to Lutz et al. (2008, p. 163), meditation is defined as ‘a family of complex emotional and attentional regulatory training regimes developed for various ends, including the cultivation of well‐being and emotional balance’. The authors distinguish between two meditation styles that have been most commonly studied: focused attention meditation and open monitoring meditation. In focused attention meditation, practitioners direct their attention towards a chosen object, whereas open monitoring meditation ‘involves nonreactive monitoring of the content of experience from moment to moment’ (Lutz et al., 2008, p. 163). Dahl et al. (2015) later expanded this framework by proposing three meditation families based on their primary cognitive mechanisms: attentional, constructive and deconstructive practices.

The attentional family is defined as ‘a class of meditation practices that strengthen the self‐regulation of various attentional processes, especially the ability to initiate and sustain meta‐awareness’ (Dahl et al., 2015, p. 515). They further describe meta‐awareness as the ‘cognitive function of being aware of the processes of consciousness’ (Dahl et al., 2015, p. 516). From this perspective, both focused attention and open monitoring meditation, as described by Lutz et al. (2008), can be considered part of the attentional family.

The constructive family, in turn, comprises ‘meditation practices that allow one to cultivate, nurture, or strengthen cognitive and affective patterns that foster well‐being’ (Dahl et al., 2015, p. 515). The authors identify perspective taking and cognitive reappraisal as central processes within this family. Extending this account, Engen and Singer (2016) highlighted the self‐generation of motivational and emotional states as another important process within constructive practices. Finally, the deconstructive family ‘uses self‐inquiry to foster insight into the processes of perception, emotion, and cognition’ (Dahl et al., 2015, p. 516).

Compared with the broader evidence base on resilience interventions in adults, systematic reviews examining meditation as a resilience intervention for university students remain relatively scarce. In their integrative literature review, Van der Riet et al. (2018) found meditation to be beneficial for mental health and potentially also for resilience among nurses and nursing students. Similarly, the meta‐analysis by Chen et al. (2023) on mobile mindfulness meditation in university students reported significant effects on mental health‐related outcomes, including stress, anxiety, well‐being and the resilience‐related factor of mindfulness with small to moderate effect sizes. However, they did not find a significant effect on resilience itself. Comparable results were reported by Dawson et al. (2020), who found that mindfulness‐based interventions improved distress, anxiety, depression, well‐being, rumination and mindfulness in university students. But again, these effects were generally small to moderate in magnitude, and no significant improvement was observed for resilience. They suggested that this null finding may be attributable to substantial heterogeneity across studies. In line with this, the meta‐analysis by Abulfaraj et al. (2024) concluded that mindfulness‐based interventions, including meditation, did not produce significant improvements in measured resilience, despite reducing stress and anxiety.

Taken together, these findings indicate a gap in the evidence base regarding the effects of meditation on resilience in university students, with between‐study heterogeneity representing a likely challenge. This, in turn, points to the need for a more comprehensive meta‐analysis based on a clearly defined conceptualisation of meditation. One possible source of heterogeneity may lie in the presence of moderating factors. For example, Khoury et al. (2017), Dawson et al. (2020) and Parsons et al. (2017) found no significant effects of meditation style, delivery mode and intervention duration on surrogate resilience outcomes, although some of these factors were associated with adherence to the interventions (see Section 4 for details). To strengthen the evidence base, further meta‐regressions and subgroup analyses are needed. In addition, meditation has not yet been compared with other interventions within a comprehensive meta‐analytic framework, such as network meta‐analysis. Such an approach would allow more precise comparisons across multiple interventions and help to position meditation more clearly within the broader field of interventions aimed at enhancing resilience.

To date, we are not aware of any published systematic review with a network meta‐analysis that has examined the effects of meditation as a stand‐alone intervention primarily targeting psychological resilience in university students. Accordingly, the present study addresses the following research questions:

1. Do meditation‐only interventions without physical activity improve psychological resilience in university students compared with active or inactive control conditions?
2. Does the effectiveness of meditation‐only interventions without movement differ according to the type of meditation, intervention intensity (i.e. session duration, session frequency and overall programme duration), mode of delivery or length of follow‐up?
3. How does the effectiveness of meditation‐only interventions for improving resilience compare directly with that of other resilience‐ or mental health‐promoting interventions?

## METHOD

We designed and implemented a structured search, retrieval, screening and data extraction process to conduct a systematic review and meta‐analysis. The review protocol was prospectively registered in PROSPERO (ID CRD42022315088).

### Search strategy

Three databases were searched on 4 September 2025: MEDLINE (via PubMed), PsycINFO (via EBSCO) and Web of Science (Clarivate Analytics). No language or other restrictions were applied. The search strategy combined terms related to university students with terms related to meditation. Within each of these two concept groups, synonymous search terms were combined using the Boolean operator OR (e.g. meditat* [Title/Abstract] or meditation [MeSH Terms] for PubMed). The two concept groups were then combined using AND to identify studies at the intersection of meditation and university students. The remaining PICO criteria listed below were not incorporated into the search strategy in order to avoid inadvertently excluding potentially relevant studies. Full details of the search strategy are provided in Appendix S1. Duplicates were removed using both the Deduplicator Tool (Institute for Evidence‐Based Healthcare, Bond University, 2024) and the automatic deduplication feature of the Covidence software (Cleo et al., 2019).

### Inclusion and exclusion criteria

The inclusion criteria were defined as follows. First, studies had to include participants who were university students or individuals enrolled in post‐secondary education leading to an academic degree. Second, the intervention had to involve meditation, as defined by Dahl et al. (2015) or Lutz et al. (2008). Third, eligible studies had to include either inactive or active control groups (e.g. waitlist/no intervention controls or alternative interventions), and only randomised controlled trials were considered. Fourth, the outcomes of interest comprised resilience, mental health‐related constructs and any adverse events occurring during the intervention. For resilience, both trait‐ and outcome‐oriented definitions were accepted. This broader approach was chosen because of the limited evidence base for outcome‐oriented conceptualisations of resilience. Mental health‐related outcomes included anxiety, depression, stress and well‐being, quality of life or flourishing. Given the broad conceptualisation of resilience adopted in this review, these constructs were included as indicators that may also reflect students' resilience. In addition, 23 resilience factors were included as secondary outcomes, based on Kunzler et al. (2020) and further literature supporting individual constructs. These were acceptance (Yi‐Frazier et al., 2010), active coping, cognitive flexibility, cognitive control (Sasse et al., 2014), coping flexibility, creativity (Elisondo, 2021), empathy (Lietz, 2011), hardiness, hope, humour, (internal) locus of control, meaning in life or purpose in life, mindfulness (Yang & Oka, 2022), optimism or positive attributional style, positive affect, positive emotion, religiosity, spirituality or religious coping, self‐acceptance (Pilipenko, 2020), self‐compassion (Austin et al., 2023), self‐efficacy, self‐esteem, sense of coherence and social support. These outcomes were included for the same reason as the mental health‐related constructs, namely, to capture a broader range of indicators relevant to resilience. This approach is consistent with the broader conceptualisation of resilience used by Kunzler et al. (2020). Finally, studies were included only if the full text was available in English or German, as these were the languages in which the authors were able to assess the articles.

The exclusion criteria related to population, intervention, outcome and study design. First, studies including only students with existing health problems were excluded because such samples were considered insufficiently representative of the general student population. Second, interventions were excluded if they combined meditation with physical activity, such as Yoga, Tai‐Chi, Qigong or Jacobson's Progressive Muscle Relaxation (McCallie et al., 2006). Interventions involving subsequent journaling, defined as writing about the meditation experience before the post‐test assessment, were also excluded. These restrictions were applied to maintain a narrower focus and to minimise the influence of effects potentially attributable to physical activity or additional intervention components rather than meditation itself. A special case concerned the Headspace app: Although the full version includes walking meditation (Headspace, n.d.), studies using only the test version (i.e. 10 free sessions), which did not include walking meditation, were considered eligible. Interventions that combined meditation with other treatments, such as group discussion or additional mind–body practices (e.g. Mindfulness‐Based Stress Reduction; Woods & Rockman, 2021), were also excluded in order to isolate the effects of meditation as clearly as possible. Third, with regard to outcomes, studies assessing constructs that deviated substantially from the predefined outcomes were excluded. For example, to reduce heterogeneity within the anxiety outcome, studies focusing on specific forms such as test anxiety were not included. In terms of study design, studies were excluded if they did not assess outcomes at both pre‐ and post‐intervention time points. In addition, studies were excluded if they included measures that differed substantially from the quantitative questionnaire‐based assessments of interest (e.g. cognitive tasks or structured daily activities) between the relevant pre‐ and post‐intervention assessments, as these may have introduced bias. An exception was made for tasks intended to induce stress in order to assess resilience in response to a stressor. Finally, studies were excluded if they applied highly selective inclusion or exclusion criteria that substantially limited generalisability, such as restricting participation to users of iOS devices.

### Screening and data extraction

The database searches yielded 10,738 records in total, of which 7530 remained after the removal of duplicates and automatically removing non‐RCT entries by the corresponding feature of Covidence (Cleo et al., 2019). These records were imported into Covidence (Cleo et al., 2019), which was used for the subsequent screening and data extraction processes in order to reduce the risk of reviewer error.

The screening process consisted of two stages: titles and abstract screening, followed by full‐text screening. In the first stage, titles and abstracts were screened to exclude records that clearly did not meet the eligibility criteria. In the second stage, full texts were assessed to identify and exclude any remaining ineligible studies. Where possible, unavailable full texts were obtained through the library of the University Medical Center Mainz. Both title/abstract screening and full‐text screening were conducted independently by at least two reviewers who were blinded to each other's decisions. For studies that were extracted in parallel, reviewer agreement during data extraction was high (*N* = 36). Therefore, data extraction for seven studies was verified by a second reviewer, whereas the remaining studies (*N* = 45) were extracted by a single reviewer. The same procedure was applied to the risk of bias assessment. The reviewers involved in these processes were RJ, TD, PK, JR and VE. At the end of each stage, discrepancies were resolved through discussion and agreement among the reviewers. Interrater reliability, including proportional agreement and Cohen's kappa (κ), was calculated using Covidence. Missing data were requested from study authors by email. If no response was received within 1 to 2 weeks, a reminder email was sent.

The remaining studies proceeded to the data extraction stage. At this stage, all relevant data and text passages were extracted using a previously developed extraction form. The form was piloted on the first 10 studies and subsequently refined to better reflect the structure of the included studies. In parallel with data extraction, risk of bias was assessed using the Cochrane Risk of Bias 2 tool (RoB2; Sterne et al., 2019). To support this assessment, all available study protocols and preregistrations were examined to identify any discrepancies between the planned and actual conduct of the studies. To further reduce reviewer error, the RoB2 Excel tool was used, as it provides automated decision algorithms and background guidance for each signalling question (riskofbias.info, 2019).

### Data synthesis

Once data extraction and risk of bias assessment had been completed, all extracted study data were transferred from Covidence to Jupyter Notebook, which was used to run the *R* programming language (see Appendix S2 for the analysis code and the corresponding libraries and versions). All data processing and statistical analyses were conducted exclusively through these R scripts, without any manual data transfer or manipulation following data extraction. This approach was chosen to ensure full traceability of all calculations and to minimise the risk of inadvertent analytic errors. The numbers reported in the flow chart of the search and screening process were generated automatically by Covidence. For all significance tests, the threshold for statistical significance was set at *p* ≤ 0.05. No prospective (a priori) power analyses were conducted for the analyses reported in the following two subsections. Retrospective (post hoc) power analyses were not performed because their usefulness remains controversial (Wang, 2010). All analysis code was written by the first author (RJ).

### First research question (effect of meditation on resilience) and third research question (comparison to other interventions)

The first research question, concerning the effect of meditation on resilience, was examined by fitting random‐effects models to between‐group post‐test outcomes comparing meditation interventions with passive control conditions. These models were estimated using the *meta* package in R with its default calculation settings. Pooled effect estimates, corresponding 95% confidence intervals (CIs), and *p* values were calculated. The standardised mean difference (SMD; Hedges' *g*) was used as the effect size measure. Following Cohen (1988), SMDs were interpreted using conventional thresholds, with values of approximately 0.2, 0.5 and 0.8 indicating small, medium and large effects, respectively. Effect sizes were calculated from post‐intervention means, standard deviations and sample sizes. In some studies, it was unclear whether the reported sample sizes referred to pre‐ or post‐intervention assessments. As these studies also did not report imputation procedures, post‐intervention sample sizes were assumed. No conversions to Hedges' *g* were necessary, as no study with sufficient data for SMD calculation reported an alternative effect size (e.g. Cohen's *d*). Missing data were not imputed. Accordingly, studies lacking the necessary data were excluded from the analyses. Some included studies used multi‐arm designs, comparing more than one meditation condition with the same control group. The resulting dependence between effect sizes was not modelled explicitly, as the number of such cases was small. The restricted maximum‐likelihood estimator was used to estimate the amount of between‐study heterogeneity $\tau^{2}$ (Viechtbauer, 2005). In addition, Cochran's *Q* and the *I* ^2^ statistics were calculated. In line with the preregistered protocol, *I* ^2^ values greater than 50% were interpreted as indicating substantial heterogeneity.

In addition to the planned outcome‐specific analysis, random‐effects network meta‐analyses were conducted using a frequentist weighted least squares approach. Separate models were estimated for each of the outcome domains described in Section 1: (1) resilience scales, (2) resilience‐related mental health outcomes (e.g. anxiety, depression, stress and well‐being) and (3) resilience factors or secondary outcomes (e.g. mindfulness, hope, self‐compassion, self‐efficacy and active coping). This approach was chosen to derive an overall estimate for each outcome domain. In addition, the network meta‐analyses included all available active and passive control conditions, allowing the comparison of meditation with a broader range of comparators, such as physical rest or cognitively engaging control conditions. This enabled us to examine whether the standardised mean difference for meditation relative to passive control conditions varied according to the nature of the comparison condition, such as physical inactivity or specific cognitive activation. The network meta‐analyses were conducted using the *netmeta* package in R with default settings, except for the tolerance threshold for inconsistency in multi‐arm studies. Because some multi‐arm studies showed a higher degree of within‐study inconsistency than permitted under the default setting in *netmeta*, this threshold was relaxed to allow their inclusion in the model. The impact of this decision was examined further in the sensitivity analyses.

### Second research question (influence of moderators)

To address the second research question concerning the influence of moderators, mixed‐effects meta‐regression models were fitted based on between‐group post‐test comparisons. These analyses were conducted using the *metafor* package in R with its default settings. The continuous moderators examined were programme duration (days), session duration (minutes), session frequency (sessions per week) and follow‐up length (days). For categorical moderators, subgroup analyses were conducted using mixed‐effects models with separate within‐group $\tau^{2}$ estimates. The categorical moderators were delivery mode (synchronous and asynchronous) and meditation family (attentional, constructive, deconstructive and hybrid). For the moderator delivery mode, classification was based on whether the provision and receipt of meditation instructions occurred simultaneously. For instance, an audio‐recorded meditation session was classified as asynchronous. Consistent with the registered protocol and the recommendation of Deeks et al. (2023), analyses were not conducted when fewer than 10 studies were available, as such analyses are considered unreliable with small numbers of studies. Accordingly, studies were required to report, at minimum, the mean, standard deviation and sample size for both intervention and control groups at post‐intervention, as well as the relevant moderator data. For an analysis of follow‐up length, studies additionally had to report these data for the post‐intervention assessment and at least one subsequent follow‐up time point. As in the analyses addressing the first research question, missing data were not imputed, studies lacking the required information were excluded. As noted above, a network meta‐analysis including all outcomes was conducted using *netmeta*. However, because this package does not support network meta‐regression, moderator analyses were not performed within the network meta‐analytic framework.

### GRADE approach

The strength of the body of evidence was assessed using the Grading of Recommendations Assessment, Development and Evaluation (GRADE) approach (Schünemann et al., 2013). The corresponding Summary of Findings table was generated by the GRADEpro software. In contrast to the original protocol, no separate table was created for individual meditation families, as no robust differences were identified between them.

## RESULTS

### Study sample and design characteristics

**FIGURE 1:**
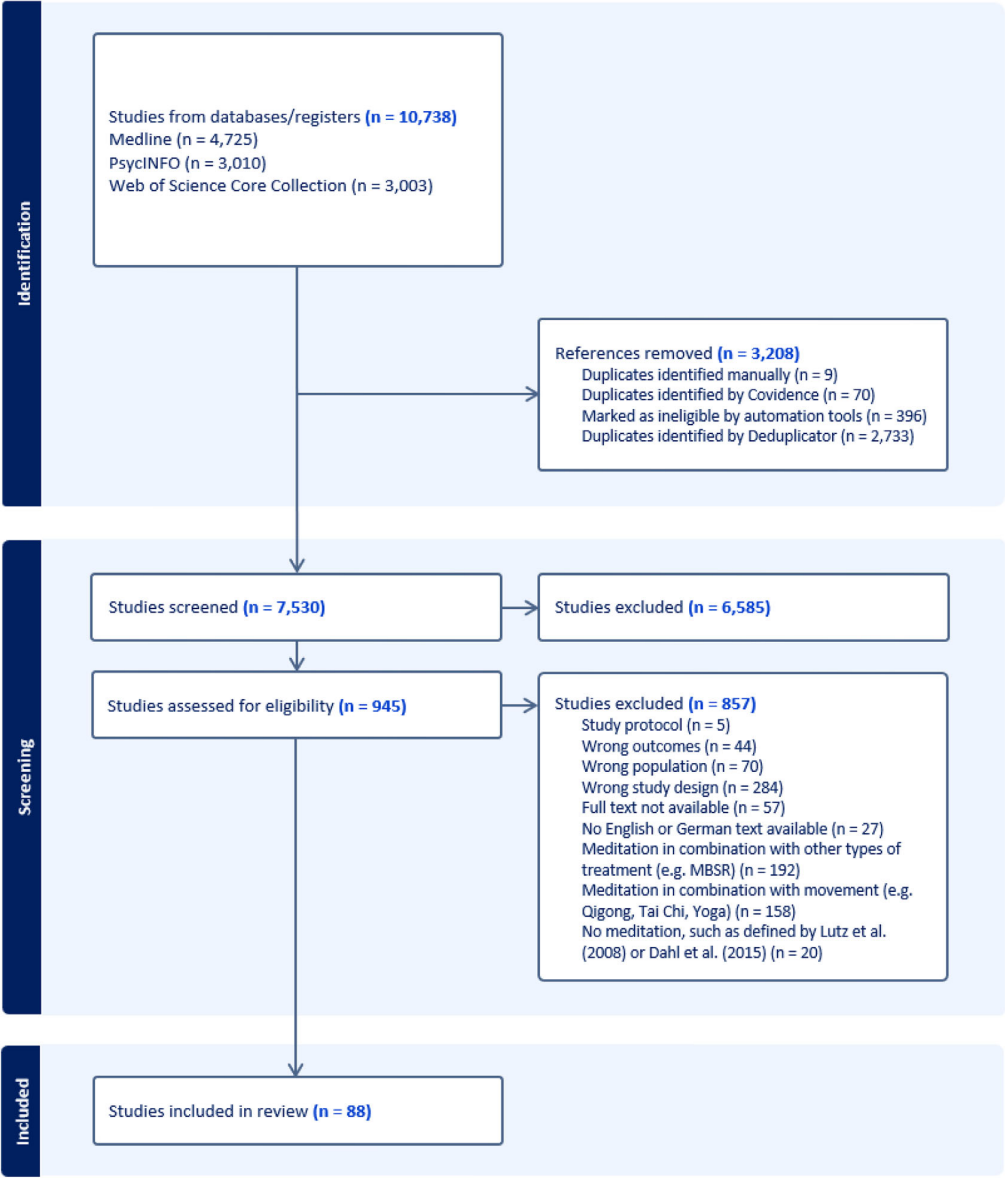
Flow chart of the search and screening process.

Interrater reliability was high, with proportional agreement ranging from 0.90 to 0.93 and Cohen's kappa ranging from 0.48 to 0.62. As shown in the PRISMA flow diagram (Figure 1), a final sample of 88 studies met the eligibility criteria and was included in the present review. Across these studies, SMDs were calculated for 207 pairwise comparisons between intervention and control groups spanning 10 different treatments. In total, the included studies comprised 8728 participants, with missing sample sizes imputed using the overall mean. The United States was the most frequently represented study location, accounting for 44 studies. All other regions were represented by between one and six studies.

The distribution of meditation families was dominated by the attentional family, which occurred 98 times, followed by the constructive family (21 occurrences) and the deconstructive family (6 occurrences). The total number of family occurrences exceeded the total number of included studies because some interventions combined elements from more than one meditation family. In such cases, each family represented within an intervention was counted separately. In addition, meditation families were counted at the intervention level, and some studies included more than one intervention. To further characterise the interventions, descriptive statistics were calculated for overall intervention duration, as well as for the duration and frequency of individual sessions. Nearly all studies (96.59%) reported overall intervention duration. Reporting was less complete for session duration (80.68%) and session frequency (71.59%). On average, the interventions lasted 21.42 days (*SD* = 27.17, range = 0–152). Participants completed an average of 3.25 sessions per week (*SD* = 4.18, range = 0.71–33), with a mean session duration of 21.24 min (*SD* = 18.35, range = 3–120). A detailed overview of individual study characteristics is provided in Appendix S3.

The outcomes were not evenly distributed across studies. Anxiety, stress, mindfulness and depression were the most frequently assessed outcomes (see Appendix S4). These were also the only outcomes for which at least 10 studies comparing meditation with passive control conditions provided sufficient data for meta‐analysis, which was the prespecified threshold for conducting meta‐regression and subgroup analyses.

**FIGURE 2:**
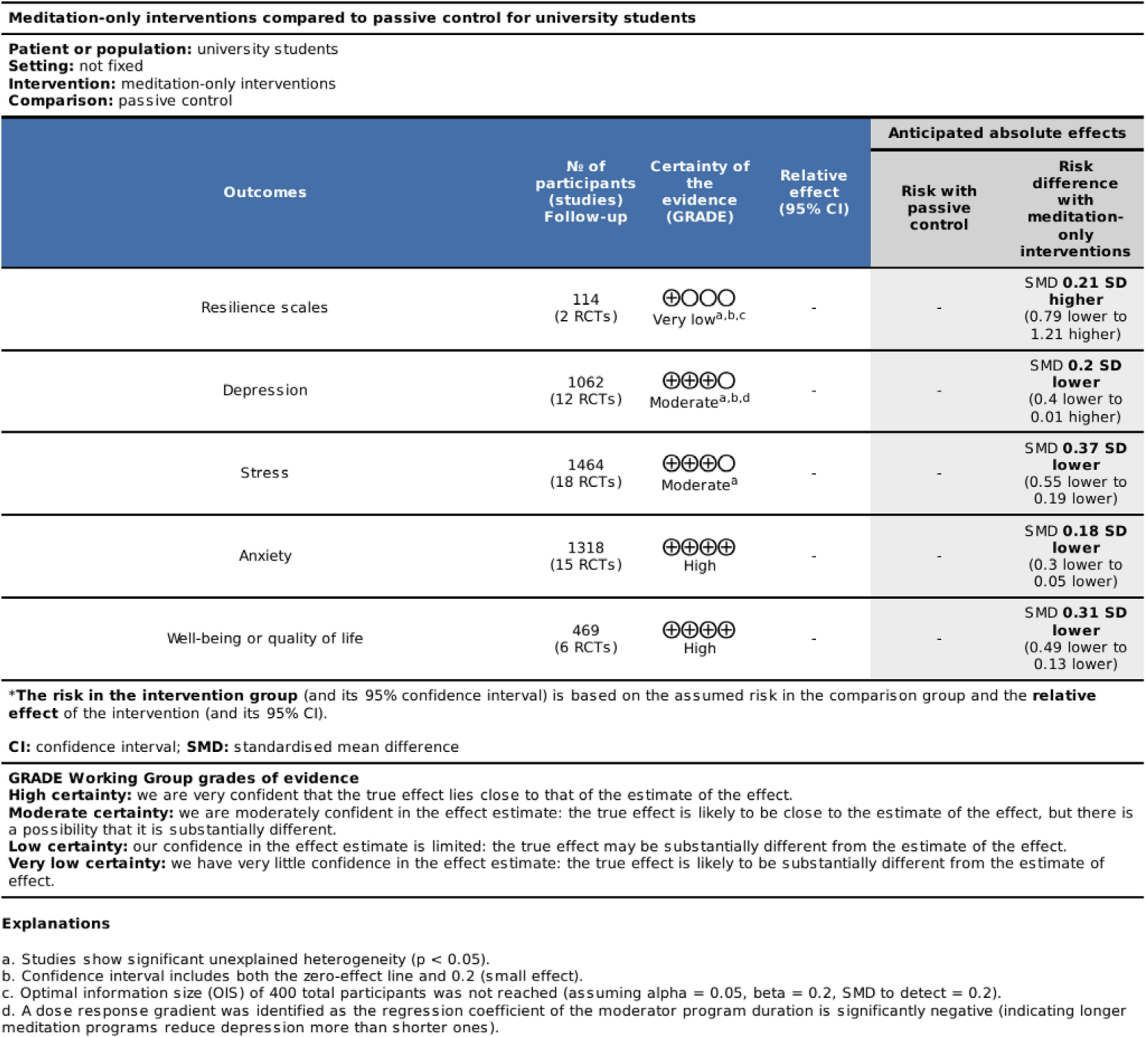
Summary of findings table according to the Grading of Recommendations, Assessment, Development and Evaluation (GRADE) working group illustrates the certainty of the evidence by a standardised method (Schünemann et al., 2013; see footnotes for explanations of rating).

Meta‐analytic decisions necessarily involve several analytical choices, including whether to apply fixed‐effect or random‐effects models and whether to retain or exclude outliers or influential cases. For all results reported in this and the following section (primary and secondary outcomes), we present the estimates based on the analytical specifications considered most appropriate by the review authors, which are coded as 1 in the sensitivity analysis tables in Appendix S17 and subsequent appendices. Alternative analytical choices are coded as 0 in the corresponding tables. To maintain the focus, the full sensitivity analyses are reported in the appendices. However, their principal implications are summarised alongside the results presented below. The summary of findings table based on the GRADE approach is presented in Figure 2. The reasons for downgrading the certainty of evidence are described separately for each outcome below.

### Primary outcomes

**FIGURE 3:**
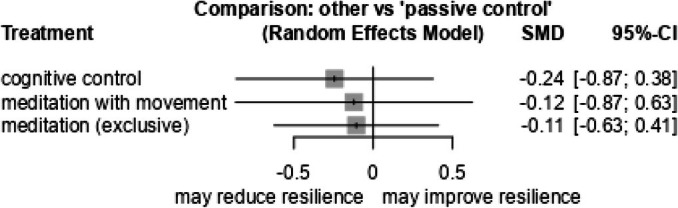
Summarising forest plot for all outcomes of the meta‐analyses comparing with passive control groups at post‐intervention timepoints (the vertical grey dotted lines show SMD = ±0.2, 0.5 and 0.8).

Table 1 summarises the results of the meta‐analyses, and Figure 3 presents a forest plot of the pooled SMD estimates for each outcome. In addition to the overall effect estimates, results are also reported separately by delivery mode (synchronous vs. asynchronous guidance) and by meditation family, distinguishing between constructive, deconstructive, attentional and hybrid forms where the criteria specified in the Methods section were met.

**TABLE 1:** Results of pairwise meta‐analyses (compared to passive controls) of between‐group post‐intervention time points for outcomes that have 10 or more included studies reporting sufficient data for meta‐analyses.

	Hedges' *g*	95%CI	*p* value	Int *n*	Con *n*	Total *n*	*Q*	*I* ^2^	*K*	Grade	PB
Anxiety overall	−0.18	−0.3 – −0.05	.01	661	657	1,318	18.87	25.8	15	High	No
Synchronous guiding	−0.42	−0.65 – −0.18	0	137	147	284	0.33	0	4	–	–
Asynchronous guiding	−0.09	−0.28 – 0.09	.31	386	412	798	13.3	32.32	10	–	–
Constructive family	–	–	–	–	–	–	–	–	–	–	–
Attentional family	−0.2	−0.33 – −0.07	0	469	447	916	7.18	0	12	–	–
Hybrid form	−0.01	−0.55 – 0.53	.97	192	210	402	11.19	82.13	3	–	–
Depression overall	−0.2	−0.4 – 0.01	.06	536	526	1,062	27.2	59.56	12	Moderate	No
Synchronous guiding	−0.29	−0.61 – 0.03	.07	143	158	301	5.41	44.55	4	–	–
Asynchronous guiding	−0.23	−0.5 – 0.05	.11	255	270	525	12.8	53.12	7	–	–
Constructive family	−0.72	−1.18 – −0.25	0	35	42	77	0	–	1	–	–
Attentional family	−0.2	−0.41 – 0.01	.07	386	360	746	14.66	45.45	9	–	–
Hybrid form	0.09	−0.57 – 0.74	.8	115	124	239	5.75	82.61	2	–	–
Stress overall	−0.37	−0.55 – −0.19	0	726	738	1,464	42.16	59.68	18	Moderate	No
Synchronous guiding	−0.51	−0.74 – −0.27	0	142	159	301	0.22	0	4	–	–
Asynchronous guiding	−0.37	−0.61 – −0.13	0	446	481	927	33.55	64.23	13	–	–
Constructive family	−0.59	−1.05 – −0.13	.01	35	42	77	0	–	1	–	–
Attentional family	−0.39	−0.58 – −0.19	0	505	491	996	25.08	52.15	13	–	–
Hybrid form	−0.28	−0.79 – 0.23	.29	186	205	391	15.57	80.73	4	–	–
Mindfulness overall	0.82	0.2–1.43	.01	437	479	916	126.8	92.11	11	‐	No
Synchronous guiding	0.38	0.1–0.67	.01	86	109	195	0	–	1	–	–
Asynchronous guiding	0.86	0.18–1.54	.01	351	370	721	123.21	92.7	10	–	–
Constructive family	–	–	–	–	–	–	–	–	–	–	–
Attentional family	0.87	0.12–1.62	.02	367	401	768	121.67	93.42	9	–	–
Hybrid form	0.6	−0.16 – 1.36	.12	70	78	148	5.13	80.49	2	–	–

Abbreviations: Int *n* and Con *n*, participants in the intervention and control group; K, included studies; PB, publication bias detected.

#### Resilience scales

The pooled SMD for resilience scale outcomes did not differ significantly from zero (95% CI = −1.21 to 0.79). Owing to the wide confidence interval, substantial unexplained heterogeneity and the small number of participants, the certainty of the evidence was downgraded to very low according to the GRADE approach.

#### Anxiety

For Anxiety, the pooled SMD was −0.18, indicating a small effect, and differed significantly from zero (95% CI = −0.30 to −0.05). The certainty of the evidence for this outcome was rated as high. Subgroup analyses revealed no significant differences across either meditation family or delivery mode.

#### Depression

For depression, the pooled SMD was −0.20 and did not significantly differ from zero (95% CI = −0.40 to 0.01). The certainty of evidence for this outcome was downgraded to moderate because of the following reasons: (1) significant unexplained between‐study heterogeneity, (2) 95% CI crossing both 0 and 0.2 (small effect) and (3) a significant dose‐response gradient was found.

#### Stress

The pooled SMD for stress was −0.37, indicating a small‐to‐moderate effect, and differed significantly from zero (95% CI = −0.55 to −0.19). The certainty of the evidence for this outcome was rated as moderate because of unexplained between‐study heterogeneity.

#### Well‐being

For well‐being, the pooled SMD was 0.31, indicating a small‐to‐moderate effect, and differed significantly from zero (95% CI = 0.13 to 0.49). The certainty of the evidence for this outcome was rated as high.

#### Adverse events

No adverse events were reported across the included studies.

### Secondary outcomes

The pooled SMDs for the secondary outcomes' mindfulness, hope, positive emotion and self‐compassion differed significantly from zero with effects ranging from moderate to large in favour of resilience. By contrast, the pooled estimates for the other secondary outcomes (self‐efficacy, active coping, optimism, empathy and self‐acceptance) did not differ significantly from zero.

### Moderation effects

#### Anxiety

With regard to the subgroup analyses for anxiety, the initial model suggested a significant difference between synchronous and asynchronous delivery of meditation interventions. However, this subgroup difference was not robust in the sensitivity analyses. No significant subgroup differences were found for meditation family in either the primary or sensitivity analyses.

The meta‐regression analyses for anxiety indicated no significant moderation by programme duration, session duration and session frequency. No studies assessing anxiety included follow‐up measurements beyond the post‐intervention assessment. Consequently, no meta‐regression could be conducted for follow‐up length.

#### Depression

For depression, neither meditation family nor delivery mode showed significant subgroup differences. However, there was a significant moderation found for programme duration indicating longer meditation interventions reduced depression more than shorter ones. Although this moderation was very weak (regression coefficient = −0.01, 95% CI = −0.02 to 0.00), it was found to be robust in all sensitivity analyses. The other continuous moderators for depression were non‐significant.

#### Stress

For stress, no significant subgroup differences were found for either meditation family or delivery mode. Likewise, none of the moderators examined in the meta‐regression analyses showed a significant effect. However, programme duration and session duration emerged as significant moderators in the sensitivity analyses after exclusion of outlying studies. As the 95% CIs of the regression coefficients cross zero for both moderators, the direction of this moderation remains unclear.

#### Mindfulness

Among the secondary outcomes, only mindfulness met the threshold of at least 10 studies required for both subgroup analyses and meta‐regression. Neither meditation family nor delivery mode showed significant subgroup differences. However, the subgroup distributions were highly imbalanced, which may have limited the accuracy of these results. The follow‐up period was the only continuous moderator with adequately reported data to permit meta‐regression analysis. However, this moderator did not reach statistical significance.

### Network meta‐analysis with all outcomes

The following sections report the results of the network meta‐analysis.

#### Resilience scales

**FIGURE 4:**
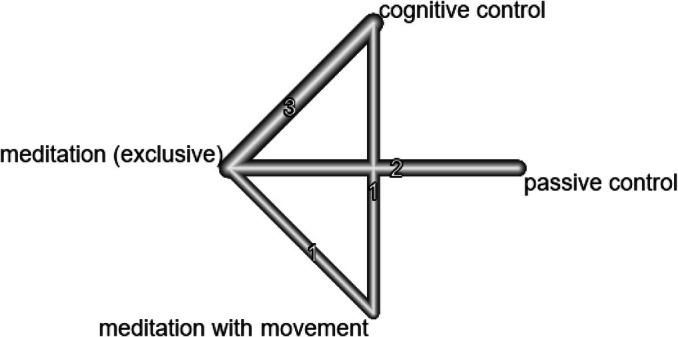
Forest plot for the network meta‐analysis model including resilience scales as outcome (each row shows the respective treatment's SMD and its 95% CI relative to the passive controls).

The random‐effects network meta‐analysis for resilience scale outcomes yielded an overall SMD of −0.11 (95% CI = −0.63 to 0.41, *p* = .68), indicating no significant difference between meditation‐only interventions and passive control conditions. As shown in Figure 4, the model also indicated no significant difference between meditation‐only interventions and either cognitive‐control interventions or meditation combined with movement. However, as outlined below, the validity of this model should be interpreted with caution.

The model was based on four studies and included data from 498 observations (participants per treatment per outcome). Across these studies, SMDs were calculated for seven pairwise comparisons involving four treatments (see Table 2). Examination of heterogeneity and inconsistency suggested limited model validity. Specifically, the heterogeneity *Q* statistic was significant (*p* < .05); the *I* ^*2*^ value was 59%, exceeding the prespecified threshold of 50% for substantial heterogeneity; and the total *Q* statistic approached significance (total: *χ* ^2^ = 7.36, *df* = 3, *p* = .06; within‐design heterogeneity: χ^2^ = 7.17, *df* = 2, *p* = 0.03, between‐design inconsistency: χ^2^ = 0.18, *df* = 1, *p* = .67). These findings suggest that the uncertainty in the model was primarily driven by heterogeneity rather than inconsistency. As part of the sensitivity analyses, the tolerance for multi‐arm study inconsistency in the netmeta() function in *R* was reduced to 0.01 and 0.001. These analyses did not identify any individual study as a source of inconsistency. However, the structure of the treatment network did not permit further assessment of heterogeneity and inconsistency using node‐splitting or net heat plot approaches. Accordingly, these additional sensitivity analyses could not be conducted for the resilience scale outcome domain.

**TABLE 2:** Overview of different interventions in the reviewed studies reporting sufficient data for meta‐analysis.

Intervention or control group classifications that delivered enough data for the meta‐analysis	Description of intervention or control group classification based on the included studies	First author's name and publication year of studies reporting sufficient data for meta‐analysis in which this intervention or control group was present
Biofeedback	Audio‐based training to enhance the EEG‐alpha amplitude at the Pz site (10–20 system)	Ratanasiripong et al., 2015
Cognitive control	Groups to control for cognitive processes such as: Exposure to media content such as nature sounds, audio tracks of poems, interviews, or news, videos that were similar to those used in the intervention just without meditation instructions Analysing poems Use of different apps to focus attention on like the Evernote app for notetaking Self‐chosen activities like reading, chatting with study colleagues, browsing the internet, or napping Cognitive exercises like listening to externally oriented statements that were unrelated to the self or finding symbols within a square matrix Deep breathing without mindfulness aspects	Gallagher, 2022; Liu et al., 2022; Flett, Hayne, et al., 2019; Flett, Fletcher, et al., 2019; Klempel, 2019; Holden, 2022; Heinrich, 2023; Heath, 2022; Holden & O'Connell, 2023; Karing, 2024; Tsai et al., 2017; Zeidan et al., 2010; Wang et al., 2021)
Dog therapy	First, ‘participants were introduced to a certified therapy dog and the dog's handler’. Afterwards, participants were left in a room with this dog.	Spruin et al., 2021
Meditation‐only interventions	Interventions that fitted the meditation definitions from the introductions (including autogenic training by Schultz)	Bultas et al., 2021; Johnson‐Waddell, 2018; Schulte‐Frankenfeld & Trautwein, 2021; Dorais & Gutierrez, 2021; Nolan, 2020; Messer et al., 2016; Tanner et al., 2009; Siembor, 2018; Huberty et al., 2019; Bonamo et al., 2015; Gallagher, 2022; Liu et al., 2022; Flett, Hayne, et al., 2019; Flett, Fletcher, et al., 2019; Klempel, 2019; Holden, 2022; Heinrich, 2023; Heath, 2022; Slater, 2015; Sloan, 2016; Weytens et al., 2014; Barry et al., 2019; Komariah et al., 2022; Waechter et al., 2021; Ratanasiripong et al., 2015; Lee & Jung, 2018; Warnecke et al., 2011; Ortiz Castro et al., 2025; Smith et al., 2021; Devillers‐Réolon et al., 2022; Archary & Thatcher, 2021; Holden & O'Connell, 2023; Karing, 2024; Kim et al., 2021; Wang & Farb, 2023; Ramsburg & Youmans, 2014; Tsai et al., 2017; Lancaster et al., 2016; Aspy & Proeve, 2017; Klibert et al., 2022; Chen et al., 2013; Grogan, 2025; Wang et al., 2021; Tloczynski, 1994; Silvestre‐López et al., 2021; Dillbeck, 1977; Zeidan et al., 2010; Shenesey, 2014; Spruin et al., 2021; de Grâce, 1976; Toole & Craighead, 2016; Vasiliauskas & McMinn, 2013
Meditation including movement	Meditation that fitted to named meditation definition but included movement like Yoga, mindful colouring or mindful eating	Flett, Fletcher, et al., 2019; Waechter et al., 2021; Karing, 2024
Mindfulness‐Based Stress Reduction (MBSR)	Intervention which included the following: mindfulness meditation, mindful attention, recalling the mind to the breath, patience, letting go and poetry reflecting mindfulness perspectives	Oman et al., 2007
Passive control groups	No intervention or wait‐list control group (participants received interventions after the study)	Bultas et al., 2021; Johnson‐Waddell, 2018; Schulte‐Frankenfeld & Trautwein, 2021; Dorais & Gutierrez, 2021; Nolan, 2020; Messer et al., 2016; Tanner et al., 2009; Siembor, 2018; Huberty et al., 2019; Bonamo et al., 2015; Sloan, 2016; Weytens et al., 2014; Barry et al., 2019; Komariah et al., 2022; Waechter et al., 2021; Ratanasiripong et al., 2015; Lee & Jung, 2018; Warnecke et al., 2011; Ortiz Castro et al., 2025; Smith et al., 2021; Devillers‐Réolon et al., 2022; Chen et al., 2013; Grogan, 2025; Wang et al., 2021; Tloczynski, 1994; de Grâce, 1976; Toole & Craighead, 2016; Vasiliauskas & McMinn, 2013
Progressive Muscle Relaxation (PMR)	Technique of repetitively tensing and relaxing muscles to reach deeper relaxation	Messer et al., 2016; Slater, 2015; Lancaster et al., 2016; Aspy & Proeve, 2017; Shenesey, 2014
Rest	Physical and cognitive inactivity such as sitting silently (most of the cases; sometimes also with instructions to close eyes or no focus on any thoughts) or waiting in a room and looking at a plant	Archary & Thatcher, 2021; Ramsburg & Youmans, 2014; Silvestre‐López et al., 2021; Dillbeck, 1977; Tloczynski, 1994
Stress management	Various combinations of interventions partially named as stress management which include psychoeducation about mechanisms of stress, relaxation techniques, self‐reflection, re‐imagining exercises of memories and emotions, positively reframing own stress and emotion regulation trainings	Weytens et al., 2014; Kim et al., 2021; Wang & Farb, 2023; Klibert et al., 2022; Spruin et al., 2021
Walking	One hour of group walking around the university campus	Waechter et al., 2021

**FIGURE 5:**
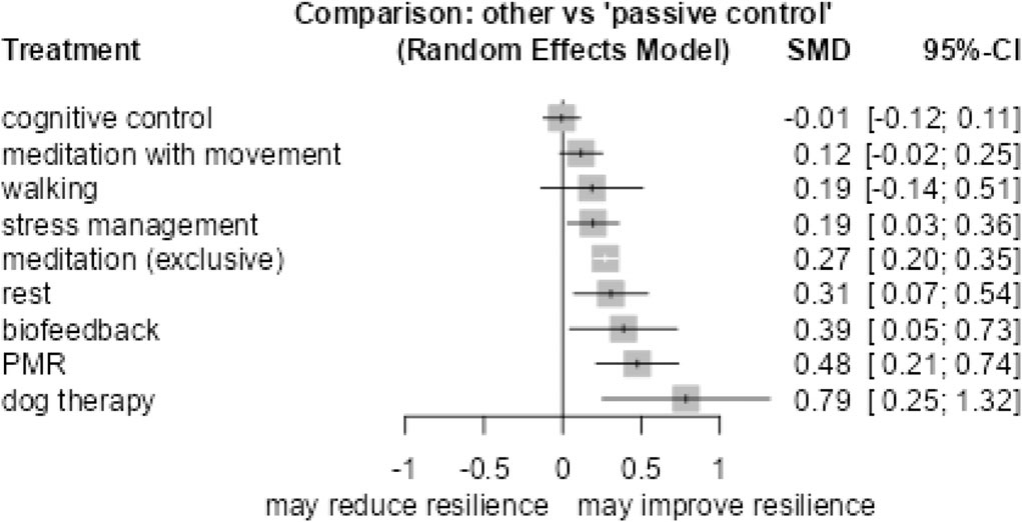
Network graph for the network meta‐analysis model of the outcome resilience scales including all present treatment groups (edges between treatments = all present direct treatment comparisons from the studies, width of the edges and numbers on the edges = number of entries that the model perceives as studies that are included for this comparison). See ‘inflated number of studies’ below.

The four included treatments are shown in the network graph in Figure 5. The edges between treatments represent the direct treatment comparison available from the included studies. In this figure, edge width usually reflects the number of studies contributing to each comparison, which is the default setting in netmeta. However, because data from some studies had to be split and treated as if they originated from separate studies within the model, the edge widths and corresponding *n* reflect this inflated count. In this case, the total was five, comprising one outcome across four studies, one of which was a split multi‐arm study. Accordingly, thicker edges and higher numbers indicate a greater number of included studies, outcomes or split multi‐arm study comparisons contributing to a given direct comparison. For descriptions of the treatment categories and their classification as cognitive control, stress management or meditation with movement, see Table 2. Within the network, the comparison between meditation‐only interventions and cognitive control was supported by the greatest amount of direct evidence (three comparisons), whereas the comparisons of meditation‐only interventions versus passive control, cognitive control versus meditation with movement and meditation‐only interventions versus meditation with movement were supported by two, one and one comparisons, respectively.

#### Resilience‐related mental health outcomes

**FIGURE 6:**
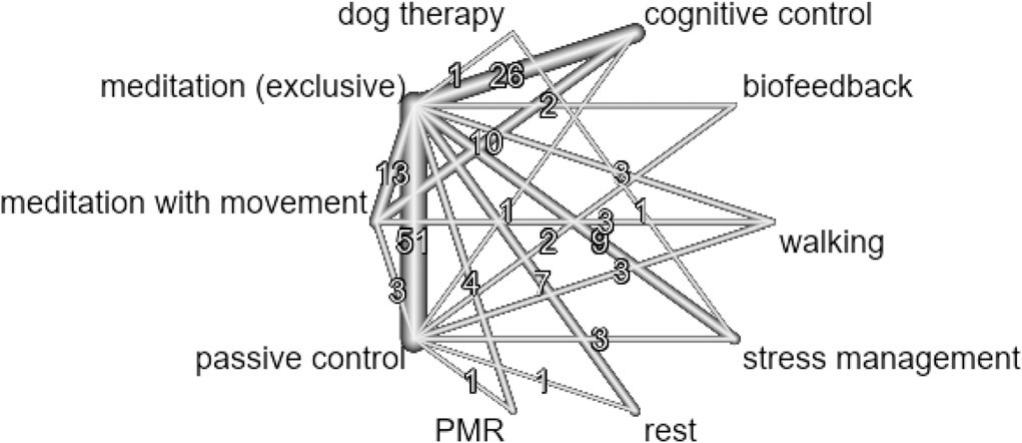
Forest plot for the network meta‐analysis model for the outcome domain resilience‐related mental health outcomes (each row shows the respective treatment's SMD and its 95% CI relative to the passive controls). PMR, progressive muscle relaxation; rest, physical inactivity.

Figure 6 presents the overall SMD estimates for the treatments included in the resilience‐related mental health domain, comprising anxiety, stress, depression and well‐being. The overall SMD for meditation‐only interventions compared with passive control groups was 0.27 (95% CI = 0.20 to 0.35, *p* < .01). Additional treatment comparisons are described in the following paragraphs. The model included 39 studies that provided sufficient data for network meta‐analysis. In total, 4536 observations from 144 pairwise treatment comparisons contributed to the model. The indicators of heterogeneity showed a mixed pattern. Although the *I* ^2^ value did not suggest substantial heterogeneity (*I* ^2^ = 40%), the *Q* statistic for heterogeneity was significant. A similar pattern was observed for inconsistency (total: *χ* ^2^ = 207.3, *df* = 124, *p* < .01; within‐design heterogeneity: *χ* ^2^ = 154.92, *df* = 102; *p* < .01, between‐design inconsistency, *df* = 22; *p* < .01). The potential sources of heterogeneity and inconsistency are explored further in the sensitivity analyses.

**FIGURE 7:**
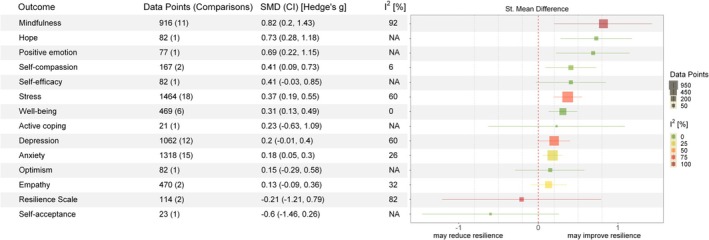
Network graph for the network meta‐analysis model of the outcome domain resilience‐related mental health outcomes (anxiety, depression, stress and well‐being) including all present treatment groups (edges between treatments = all present direct treatment comparisons from the studies, width of the edges and numbers on the edges = number of entries that the model perceives as studies which are included for this comparison). See ‘inflated number of studies’ below.

To interpret the following results, it is important to distinguish between direct and indirect evidence in network meta‐analysis (see ​Harrer et al., 2022). A further consideration in the present model is that a lower inflated study count, reflected by thinner edges in the network graph (Figure 7), likely also corresponds to a smaller number of included outcomes contributing to the respective SMD estimate. This is particularly likely when a treatment comparison is based mainly on direct evidence because, in such cases, the estimate can draw only on the outcomes observed in those direct comparisons. Put differently, comparisons represented by thin edges in the network graph are more likely to rely on a limited set of outcomes. By contrast, comparisons supported by more evidence, as indicated by wider edges in Figure 7, are more likely to include a broader range of outcomes and may therefore provide a more comprehensive representation of the resilience‐related mental health domain.

Applying the rationale outlined in the previous section to the forest plot in Figure 6, interpretation of resilience‐related mental health outcomes should focus primarily on comparisons represented by wider edges and higher counts in the network graph (Figure 7). On this basis, and assuming a causal interpretation, meditation‐only interventions appear to have a significant moderate effect on resilience‐related mental health outcomes in university students compared with passive control conditions (SMD = 0.27, 95% CI = 0.20 to 0.35, *p* < .01). A similar pattern was observed for comparisons of meditation‐only interventions with both cognitive control conditions and meditation combined with movement (see Appendix S5 for details of the sensitivity analyses).

Within the model, simple rest (physical inactivity) appeared to be similarly effective to meditation‐only interventions, as no significant difference was observed between these conditions (*p* = .76; Appendix S26). However, this comparison was based on a relatively limited evidence base, with an inflated study count of seven, derived from six studies and only half of the outcomes included in this domain (i.e. anxiety and stress). In addition, 99% of the evidence for this comparison was direct, suggesting that the estimate was driven almost entirely by these two outcomes. Accordingly, this finding provides only limited support for conclusions about the broader domain of resilience‐related mental health, although it may still be interpreted as a preliminary indication relevant to this outcome domain.

#### Resilience factors

An exploratory network meta‐analysis model for the secondary outcomes/resilience factors is presented in Appendix S5.

### Sensitivity analyses

Only the outcomes stress, mindfulness and depression reached or were very close to the prespecified threshold for substantial between‐study heterogeneity (*I* ^2^ = 50%). Overall, this suggests that most outcome estimates were unlikely to be substantially affected by between‐study heterogeneity. Moreover, the heterogeneity observed for these outcomes could not be explained by the investigated moderators or by intervention or participant characteristics.

The sensitivity analyses further indicated that both the tests of whether pooled SMDs differed from zero and the tests of between‐study heterogeneity (*Q* statistics) were generally robust across the analytical choices examined. The main exception concerned the test of the pooled SMD for depression and the *Q* statistics for depression, stress and mindfulness. In the primary analyses, the *Q* statistics for these three outcomes were significant, indicating heterogeneity, but in some sensitivity analyses, they were no longer significant. This pattern suggests that the observed heterogeneity was driven primarily by a small number of studies identified as outliers or influential cases (Barry et al., 2019; Bonamo et al., 2015; Bultas et al., 2021; Devillers‐Réolon et al., 2022; Messer et al., 2016; Ortiz Castro et al., 2025; Weytens et al., 2014). Comparable findings emerged for the overall SMD estimate in the network meta‐analysis model (see Appendix S5 for the studies identified as influential cases or as sources of heterogeneity in that model). Taken together, the sensitivity analyses largely reinforced the significance judgements from the primary analyses regarding whether the pooled SMDs differed from zero, whereas in some combinations of sensitivity‐analysis choices, it did. With regard to the continuous moderator analyses, which were conducted only for stress and anxiety, none of the moderator tests showed significance in the primary analyses. In the sensitivity analyses, programme duration and session duration emerged as significant moderators for stress in some analytical scenarios (Appendix S5). However, the 95% CI for the moderator coefficients consistently crossed zero, indicating that the direction of the moderation effect remained uncertain. Moreover, these findings were based on analyses showing significant heterogeneity, which again appeared to be driven by only a small number of studies (see Appendix S16). For depression, the moderation of programme duration was confirmed as no analytical scenario changed the corresponding significance judgements. Overall, the sensitivity analyses largely support the absence of moderation for the continuous moderators observed in the primary analyses, with the possible exception of programme duration and session duration for stress and programme duration for depression.

For the categorical moderators, only one significant subgroup difference was identified in the primary analyses, namely, for anxiety by delivery mode. However, this finding was not robust in the sensitivity analyses (see Appendix S5). Conversely, for depression, the primary analysis did indicate significance (see Appendix S5). Taken together, the sensitivity analyses largely support the absence of subgroup differences found in the primary analyses and weaken the evidence for a delivery mode effect on anxiety.

**FIGURE 8:**
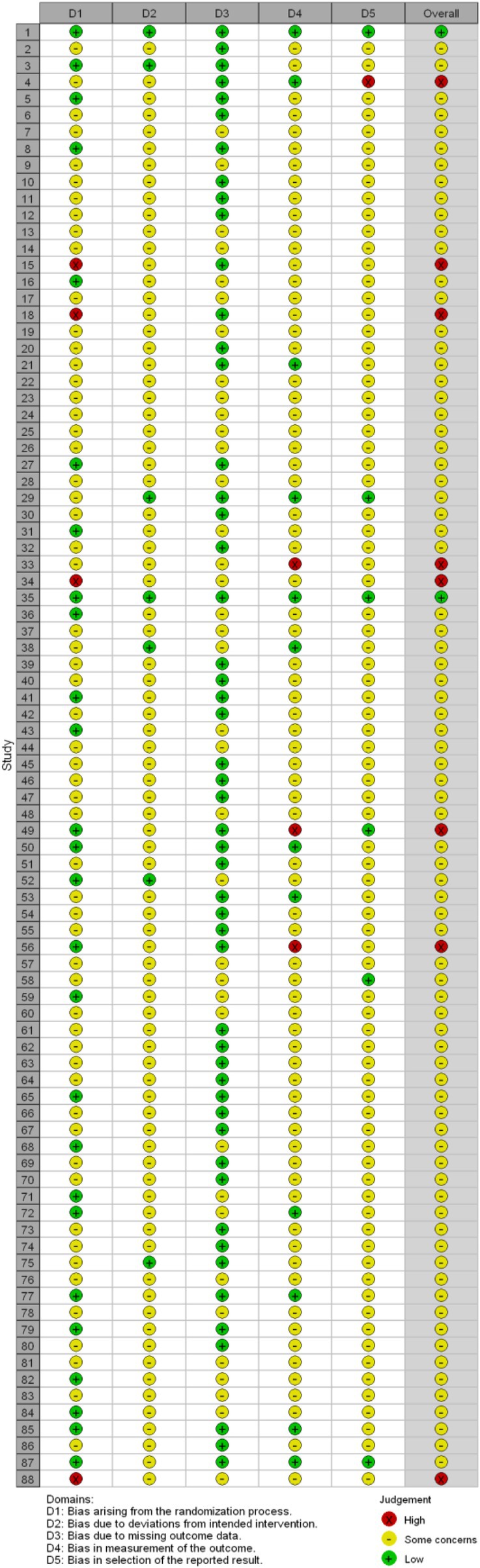
Risk of bias traffic light plot for all studies illustrating the judgement for the risk of bias (low, some concerns and high) for the respective risk of bias domain and the overall judgement according to Cochrane Risk of Bias 2 tool (Sterne et al., 2019; ID of studies corresponding to their first author and publication year as follows: 1 = (Alhawatmeh et al., 2022), 2 = (Archary & Thatcher, 2021), 3 = (Aspy & Proeve, 2017), 4 = (Barbera & Bernstein, 2025), 5 = (Barry et al., 2019), 6 = (Bonamo et al., 2015), 7 = (Bultas et al., 2021), 8 = (Chen et al., 2013), 9 = (Chow et al., 2017), 10 = (Clinton et al., 2018), 11 = (Dambrun et al., 2023), 12 = (Dawson et al., 2014), 13 = (de Grâce, 1976), 14 = (Devillers‐Réolon et al., 2022), 15 = (Dillbeck, 1977), 16 = (Dorais & Gutierrez, 2021), 17 = (Fish & Saul, 2019), 18 = (Flett, Fletcher, et al., 2019), 19 = (Flett, Hayne, et al., 2019), 20 = (Forsyth, 2017), 21 = (Gallagher, 2022), 22 = (Grogan, 2025), 23 = (Gupta & Verma, 2020), 24 = (Gutierrez et al., 2024), 25 = (Heath, 2022), 26 = (Heinrich, 2023), 27 = (Hirshberg et al., 2018), 28 = (Holden, 2022), 29 = (Holden & O'Connell, 2023), 30 = (Holland et al., 2017), 31 = (Huberty et al., 2019), 32 = (Ilies et al., 2019), 33 = (Janowiak & Hackman, 1994), 34 = (Johnson‐Waddell, 2018), 35 = (Karing, 2024), 36 = (Kim et al., 2021), 37 = (Kinney, 2022), 38 = (Kirby et al., 2021), 39 = (Klempel, 2019), 40 = (Klibert et al., 2022), 41 = (Komariah et al., 2022), 42 = (Lancaster et al., 2016), 43 = (Lee & Jung, 2018), 44 = (Liu et al., 2022), 45 = (Mantzios et al., 2021), 46 = (Messer et al., 2016), 47 = (Miller et al., 2021), 48 = (Gómez et al., 2023), 49 = (Nath & Kucukarslan, 2023), 50 = (Nidich et al., 2009), 51 = (Nolan, 2020), 52 = (Oman et al., 2007), 53 = (Ortiz Castro et al., 2025), 54 = (Ovadia‐Blechman et al., 2022), 55 = (Paholpak et al., 2012), 56 = (Philips et al., 2019), 57 = (Pilcher, Byrne, Weiskittel, et al., 2025), 58 = (Pilcher, Byrne, Wilkinson, et al., 2025), 59 = (Plummer et al., 2018), 60 = (Pogrebtsova et al., 2022), 61 = (Ramsburg & Youmans, 2014), 62 = (Ratanasiripong et al., 2015), 63 = (Rausch et al., 2006), 64 = (Sakakibara et al., 1994), 65 = (Schulte‐Frankenfeld & Trautwein, 2021), 66 = (Shenesey, 2014), 67 = (Shinde et al., 2021), 68 = (Siembor, 2018), 69 = (Silvestre‐López et al., 2021), 70 = (Slater, 2015), 71 = (Sloan, 2016), 72 = (Smith et al., 2021), 73 = (Spruin et al., 2021), 74 = (Stefanelli, 2025), 75 = (Strait et al., 2020), 76 = (Sunita et al., 2022), 77 = (Tanner et al., 2009), 78 = (Tloczynski, 1994), 79 = (Toole & Craighead, 2016), 80 = (Tsai et al., 2017), 81 = (Vasiliauskas & McMinn, 2013), 82 = (Waechter et al., 2021), 83 = (Wang et al., 2021), 84 = (Wang & Farb, 2023), 85 = (Warnecke et al., 2011), 86 = (Weytens et al., 2014), 87 = (Xu et al., 2025), 88 = (Zeidan et al., 2010).

**FIGURE 9:**
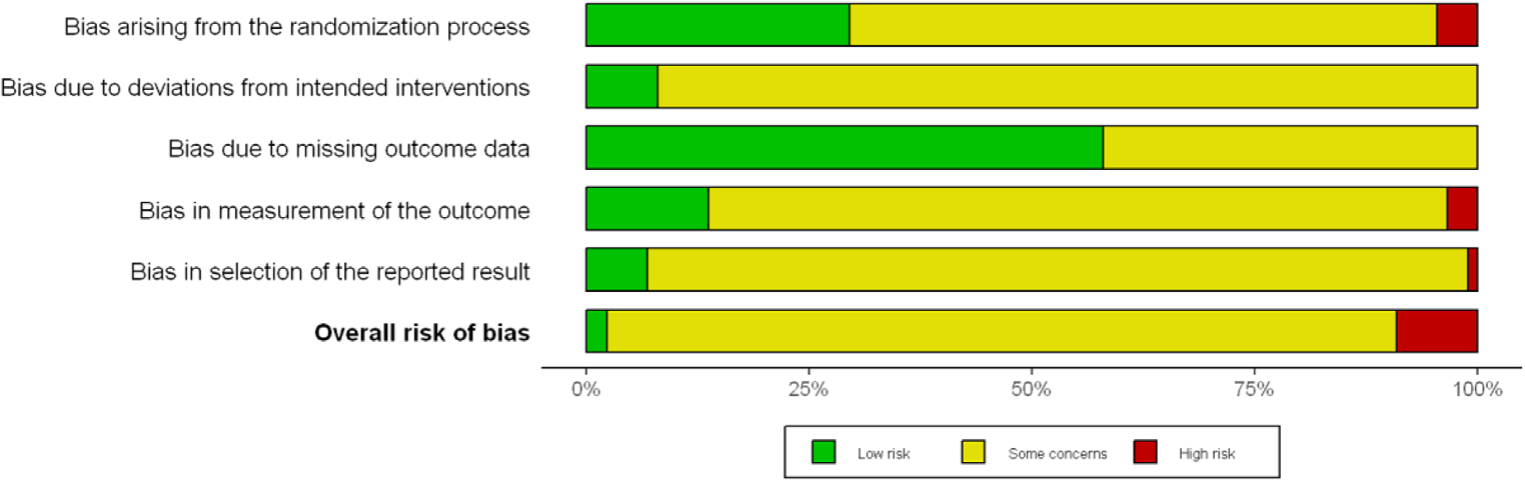
Summary bar plot for risk of bias showing the distribution of the respective levels of risk of bias for each bias domain according to Cochrane Risk of Bias 2 tool (Sterne et al., 2019).

The traffic light plot (Figure 8) indicates that Barbera and Bernstein (2025), Dillbeck (1977), Flett, Hayne, et al. (2019), Janowiak and Hackman (1994), Johnson‐Waddell (2018), Philips et al. (2019) and Zeidan et al. (2010) were rated as having overall high risk of bias according to the Cochrane Risk of Bias 2 tool (Sterne et al., 2019). All remaining studies were judged to raise some concerns, with the exception of Alhawatmeh et al. (2022), which was rated as having a low risk of bias. The distribution of these judgements across the individual bias domains is presented more clearly in Figure 9. Of the studies rated as having high risk of bias, only Johnson‐Waddell (2018) provided sufficient data for inclusion in the meta‐analysis of mindfulness. As shown in the supporting information, this study does not appear to have materially biased the corresponding results.

### DISCUSSION

The present meta‐analysis suggests that, compared with passive control conditions, meditation‐only interventions in university students were associated with an overall SMD of −0.11 (95% CI = −0.63 to 0.41) for resilience scales, 0.27 (95% CI = 0.2 to 0.35) for resilience‐related mental health outcomes and 0.54 (95% CI = 0.34 to 0.73) for resilience factors. Taken together, these findings indicate no treatment effect on resilience scales, a moderate effect on resilience‐related mental health outcomes and a moderate effect on resilience factors (first research question). However, these results should be interpreted with caution because the models were affected by heterogeneity and inconsistency. Within the network meta‐analysis, meditation‐only interventions appeared to be similarly effective to other resilience‐ or mental‐health promoting interventions, such as stress management, progressive muscle relaxation (PMR), physical or cognitive rest and biofeedback (third research question). This pattern is broadly consistent with the existing literature. In addition, we found no robust evidence for moderation by the investigated moderators, although statistical power for these analyses was limited (second research question). An exception for the latter is the moderator programme duration for the outcome depression that was found to be significantly negative (regression coefficient = −0.01, 95% CI = −0.02 to 0.00). However, this moderation was very weak. To situate these findings within the broader literature, the following sections compare the present results with previous research. We then discuss potential underlying mechanisms, practical implications and the impact of the specific inclusion and exclusion criteria applied in the present review.

#### Effects of meditation on resilience

Khoury et al. (2017) conducted a meta‐analysis on the effectiveness of traditional meditation retreats for outcomes similar to those examined in the present study, including anxiety, depression, stress, emotional regulation and quality of life. Their pooled SMD estimate (Hedges' *g* = 0.45; 95% CI = 0.35 to 0.54, *p* < .01; based on within‐group rather than between‐group analyses) was broadly comparable to the findings reported here. Similarly, Dawson et al. (2020) found that mindfulness‐based interventions improved resilience‐related surrogate outcomes in university students, including distress, anxiety, depression, well‐being, rumination and mindfulness, with small to moderate effect sizes. The beneficial effect of meditation on comparable psychological health outcomes was also supported by the review of Van der Riet et al. (2018). Taken together, the present findings for resilience‐related outcomes are broadly consistent with previous evidence syntheses, although this pattern does not extend to resilience scales themselves. According to Cohen (1988), the overall SMDs observed in the present study suggest a moderate effect for resilience‐related mental health outcomes and a moderate effect for resilience factors. Compared with other meta‐analyses of resilience‐promoting interventions, these estimates appear similar (SMD = 0.44, 95% CI = 0.23 to 0.64; Joyce et al., 2018) or in some cases larger (SMD = 0.12, 95% CI = −0.14 to 0.38; Díaz‐García et al., 2021). Meditation may therefore represent a promising intervention for promoting resilience‐related mental health outcomes and resilience factors relative to other approaches in this field. To our knowledge, the present study is the first to provide a meta‐analytic model that simultaneously compares meditation‐only interventions with passive controls, cognitive control conditions and resting control conditions. The findings suggest that meditation has a distinct effect on resilience‐related mental health outcomes. In particular, the comparison between meditation‐only interventions and cognitive control conditions was significant and indicated a moderate effect (SMD = 0.28, 95% CI = 0.19 to 0.37, *p* < .01). By contrast, no significant difference was found between meditation‐only interventions and simple rest (SMD = 0.03, 95% CI = −0.19 to 0.26, *p* = .76). One possible explanation is that resting without engaging in physical or cognitively demanding activity may overlap, to some extent, with meditation‐like states in a broader sense.

#### Moderation effects

The absence of robust moderation effects in the present review is partly consistent with previous research. For example, Khoury et al. (2017) likewise found no differences in effectiveness across meditation styles. They also reported evidence of sustained effects at follow‐up, which could not be examined in the present meta‐regression because of insufficient follow‐up data. Similarly, Dawson et al. (2020) found that intervention duration and delivery mode were not significant moderators of the effects of meditation on resilience‐related surrogate outcomes in university students. However, Parsons et al. (2017) reported that greater adherence to mindfulness‐based interventions was associated with better outcomes, including reductions in depression, anxiety and stress. Thus, the findings of the present moderator analyses are only partly consistent with the existing literature. Importantly, the lack of significant moderation effects should not be interpreted as clear evidence for the absence of true subgroup differences or moderator effects. One plausible explanation is limited statistical power. In most cases, the meta‐regressions included only slightly more than the minimum threshold of 10 studies, which restricts the ability to detect moderator effects reliably. A similar concern may apply to some non‐significant pooled SMDs. For most outcomes, the total number of participants was below 400, which would represent the optimal information size for detecting a small effect (SMD = 0.20) at alpha = .05 and beta = .20 (Schünemann et al., 2013).

#### Mechanisms underlying the effect of meditation on psychological resilience

Consistent with the present findings supporting the effectiveness of meditation for resilience‐ and mental health‐related outcomes, the mechanisms underlying these effects warrant closer consideration. Both psychological and neurobiological pathways have been discussed in the literature.

##### Psychological processes

Long‐term meditators appear to use more adaptive emotion‐regulation strategies, particularly acceptance and positive reappraisal, and to show lower levels of catastrophising. In addition, acceptance mediated the association between cortisol recovery following stress and meditation practice in a randomised controlled trial (Gamaiunova et al., 2019). Chin et al. (2019) suggested that explicit training in acceptance may be essential for achieving resilience‐related benefits. Beyond emotion regulation, attentional control has also been identified as a resilience factor against psychological distress (Ueno & Amemiya, 2024). Furthermore, self‐efficacy and self‐regulation have been discussed as additional psychological pathways through which meditation may promote resilience (Qu et al., 2022).

##### Neurobiological pathways

Functional imaging research has shown stronger connectivity between the rostral anterior cingulate cortex and dorsomedial prefrontal, praecuneus and angular gyrus following meditation, with these changes mediating both immediate and three‐month gains in resilience (Kwak et al., 2019). Calderone et al. (2024) further suggested that meditation may affect brain regions involved in emotional and sensory processing. In addition, autonomic and endocrine findings point to increased parasympathetic activity, reduced sympathetic stress reactivity, faster cortisol recovery and lower basal awakening cortisol, all of which may reflect more adaptive stress regulation (Gamaiunova et al., 2019).

In summary, these psychological and neurobiological changes may operate in a reciprocal manner: Improved emotion regulation and attentional control may promote neuroplastic adaptations, which in turn could support more efficient physiological recovery from stress. Such an integrated model may help to explain the effects observed in the present analyses.

At the same time, this interpretation should be treated with caution. The studies discussing these psychological and neurobiological mechanisms did not examine meditation‐only interventions exclusively, as defined in the present meta‐analysis, but often investigated meditation in combination with other components, such as group discussion or movement‐based practices, for example, in Mindfulness‐Based Stress Reduction (MBSR). Consequently, the associations described above cannot be attributed with certainty to meditation alone.

#### Practical implications

As noted above, the effectiveness of meditation may not differ substantially from that of other interventions aimed at improving resilience or mental health‐related outcomes. Consequently, the choice of intervention may depend largely on individual preferences. For some individuals, meditation may represent a meaningful and enriching practice, whereas for others, lower‐effort approaches, such as physical or cognitive rest without explicit mindfulness components, may be more suitable. As with other health‐promoting behaviours, meditation, as well as the alternative methods discussed above, is likely to be most beneficial when integrated into everyday life and practised regularly.

#### Limitations

##### Limitations of the present review's methods

Several methodological decisions in the present review may have affected the scope and interpretation of the findings. First, excluding movement‐based practices from the definition of meditation represented a deliberate compromise. Some practices, such as yoga, may fit our conceptual definition of meditation, but they also involve activation of the musculoskeletal system that could confound effects attributable to meditation itself. At the same time, this decision led to the exclusion of a broad range of relevant practices, including Jacobson's Progressive Muscle Relaxation (McCallie et al., 2006) and the full version of the Headspace app, which includes movement‐based meditations (Headspace, n.d.). Similarly, we excluded interventions that combined meditation with group discussion, such as MBSR (Woods & Rockman, 2021). Although these restrictions resulted in the loss of a substantial amount of potentially relevant data, they were intended to yield more precise effect estimates for meditation as an isolated intervention component.

A further limitation concerns the included outcome measures. Only self‐report measures of resilience were considered, which may underrepresent more objective indicators, such as cardiovascular or biochemical measures, that could capture additional dimensions of stress and resilience. Finally, the meta‐analyses were based exclusively on between‐group post‐test SMDs. This approach may have introduced bias in studies with shortcomings in randomisation or unaddressed baseline differences between groups. Such issues were present in at least some of the included studies.

##### Limitations of the body of literature

Only two studies included in this review, Klibert et al. (2022) and Ovadia‐Blechman et al. (2022), assessed participants' current stress load, which would have allowed evaluation of whether meditation enhances adaptation to stressful or adverse situations. More broadly, a key limitation of the current evidence base is that the review was restricted to randomised controlled trials. Although such trials offer the highest level of internal validity, this criterion excluded a substantial number of quasi‐experimental and pre–post studies examining meditation programmes. As a result, the meta‐analysis may have overlooked relevant findings, which could have influenced the overall estimate of meditation's effect on resilience. Accordingly, direct evidence on the effects of meditation on resilience remains limited. A further limitation is the reliance on surrogate outcomes, such as mental health‐related measures, rather than on direct assessments of resilience using specific resilience scales. This may have obscured the magnitude of any true resilience‐enhancing effects of meditation. In addition, several potential sources of bias were identified in the underlying literature. Some studies did not report the mode of intervention delivery, likely because face‐to‐face delivery was assumed to be standard at the time of publication. In these cases, delivery was assumed to be synchronous, which may have affected subgroup analyses. Similarly, some studies did not report the interval between baseline assessment and intervention onset or between the end of the intervention and the post‐test assessment. If these intervals were prolonged, additional bias may have been introduced.

There was also a considerable amount of missing data, particularly for the moderator analyses, which may have affected the results. In some studies, adherence was assessed using self‐report measures of meditation frequency, which are vulnerable to overestimation (Flett, Fletcher, et al., 2019). This may partly explain the ambiguous findings regarding session frequency as a moderator. Moreover, the relatively small number of studies available for many outcomes limited the validity of publication bias and heterogeneity assessments and may have contributed to underestimation of the *I* ^2^ statistic (von Hippel, 2015). For similar reasons, the resulting *R* ^2^ values should be interpreted cautiously, as López‐López et al. (2014) recommend restraint when fewer than 40 studies are included in a meta‐regression. Finally, the uneven distribution of available data across outcomes and subgroups may have further influenced the results and complicated their interpretation.

##### Limitations and strengths of the present review's emerging evidence

In summary, the evidence generated by the present analyses is limited to university student samples and to meditation‐only interventions as defined in this review. Interventions that combine meditation with other treatment components may produce different effects. Moreover, although no robust moderator (except the very weak moderation for programme duration in depression) or subgroup effects were identified, true effects may have remained undetected because of limited statistical power or the potential biases outlined above.

The main strengths and distinctive contributions of the present review are threefold. First, in contrast to much of the existing literature, we focused specifically on meditation‐only interventions. Second, we directly compared multiple interventions and resilience‐related outcomes within a single statistical model for each outcome domain, thereby extending the methodological range of the current evidence base. Third, our findings suggest that even a narrower definition of meditation, excluding movement‐based practices and combinations with other treatments, does not in itself resolve the problem of between‐study heterogeneity in indirect or surrogate estimates of resilience.

## CONCLUSION

The present study addressed three research questions: (1) whether meditation affects resilience in university students; (2) whether these effects are moderated by intervention intensity (i.e. session duration, programme duration and session frequency), delivery mode, meditation style or follow‐up period; and (3) whether meditation differs in effectiveness from other interventions in this field.

The first research question, concerning the effectiveness of meditation, was supported by the present meta‐analysis, although only with respect to resilience‐related mental health outcomes and resilience factors. Meditation‐only interventions without movement appear to be an effective approach for improving resilience‐related mental health outcomes in university students. The findings further suggest that these effects may be attributable to the cognitive processes involved in meditation and that meditation is comparably effective to other interventions in the same field. However, as discussed above, this conclusion should be interpreted with caution because it is limited by (1) the reliance on surrogate outcomes, (2) the predominance of trait‐oriented conceptualisations of resilience and (3) the absence of supporting evidence for effects on resilience scales themselves. Conclusive statements regarding moderation effects remain difficult because the available evidence is limited. In particular, only the outcomes stress, anxiety, depression and mindfulness were represented by 10 or more studies and were therefore suitable for meta‐regression. Across these analyses, no robust moderation effects (except a very weak significant negative moderation for programme duration in depression) emerged in the sensitivity analyses. There was some tentative indication that programme duration and session duration may influence stress outcomes, but this finding should be interpreted cautiously because it reached significance only in selected sensitivity analyses, such as those excluding outliers or influential cases. In addition, the direction of this moderation effect remains unclear, as the 95% confidence intervals for the regression coefficients included zero. Likewise, delivery mode (synchronous vs. asynchronous) may influence the effect of meditation on anxiety, but this finding was not robust across sensitivity analyses and therefore also requires cautious interpretation.

To address these evidence gaps, future research should examine resilience using outcome‐oriented designs that include exposure to a stressor, allowing participants' resilience to be assessed more directly. Studies on meditation should report key intervention characteristics in sufficient detail, including programme duration, session duration, session frequency and whether practice took place in a group setting or individually. For asynchronously delivered interventions, researchers should use objective measures of adherence wherever possible, such as recorded completion rates relative to the intended meditation frequency. Meditation techniques should also be described in enough detail to permit meaningful classification. In addition, future systematic reviews may benefit from broader inclusion criteria that incorporate well‐designed non‐randomised studies. This would allow a more comprehensive assessment of the field and could improve the feasibility of subgroup analyses across different study designs.

## CONFLICT OF INTEREST STATEMENT

The authors declare no conflicts of interest.

## ETHICS STATEMENT

The authors have nothing to report.

## Supporting information


**Appendix S1** Supporting Information

## Data Availability

The used R code, packages and respective versions to conduct the present network meta‐analysis are available online (https://github.com/abc4322/MA_Meta_Analyses/blob/main/Meta.Analysis%20incl%20active%20controls.ipynb).
